# A Comprehensive Review on Electrochemical Nano Biosensors for Precise Detection of Blood-Based Oncomarkers in Breast Cancer

**DOI:** 10.3390/bios13040481

**Published:** 2023-04-16

**Authors:** Mahdi Sadeghi, Somayeh Sadeghi, Seyed Morteza Naghib, Hamid Reza Garshasbi

**Affiliations:** 1 Biomaterials and Tissue Engineering Research Group, Interdisciplinary Technologies Department, Breast Cancer Research Center (BCRC), Motamed Cancer Institute, ACECR, Tehran 1517964311, Iran; 2 Department of Molecular Biology, Pasteur Institute of Iran, Tehran 1316943551, Iran; 3 Nanotechnology Department, School of Advanced Technologies, Iran University of Science and Technology (IUST), Tehran 1684613114, Iran

**Keywords:** breast cancer, electrochemical nano biosensor, oncomarkers, point-of-care-detection, breast cancer diagnosis

## Abstract

Breast cancer (BC), one of the most common and life-threatening cancers, has the highest incidence rate among women. Early diagnosis of BC oncomarkers is considered the most effective strategy for detecting and treating BC. Finding the type and stage of BC in women as soon as possible is one of the greatest ways to stop its incidence and negative effects on medical treatment. The development of biosensors for early, sensitive, and selective detection of oncomarkers has recently attracted much attention. An electrochemical nano biosensor (EN) is a very suitable option for a powerful tool for cancer diagnosis. This comprehensive review provides information about the prevalence and pathobiology of BC, recent advances in clinically available BC oncomarkers, and the most common electrochemical nano biosensors for point-of-care (POC) detection of various BC oncomarkers using nanomaterial-based signal amplification techniques.

## 1. Introduction

Cancer is typically caused by specific genetic defects in normal cells that promote tumor development and cancer cell migration. Cancerous cells often have different sizes, phenotypes, and metabolisms [1]. Meanwhile, the tumor-related microenvironment alters [2]. The cell development process is disturbed in malignant cells, creating new cells that the body does not require, and programmed cell death does not occur in these cells as it should [3]. When this happens, a buildup of cells frequently creates a tissue mass known as a lump, growth, or tumor [4]. When cancerous tumors occur in the breast, BC results, these cells can disperse by leaving the original tumor and entering lymphatic or blood channels, which branch out into other bodily regions. Cancer cells spread to other body parts through the process of metastasis, whereby they start to harm nearby tissues and organs [5].

The mortality rate of various disorders can be advantageously decreased with early diagnosis and efficient treatment choices. Depending on the available facilities, various complex physical and clinical circumstances such as magnetic resonance imaging, positron emission tomography (PET), computerized tomography (CT) scan, X-ray imaging, endoscopy, medical ultrasound, thermography, cytology, and biopsy are utilized to diagnose tumors [6,7]. Molecular techniques based on genomic and proteomic sequencing such as polymerase chain reaction, enzyme-linked immunosorbent assay (ELISA), radioimmunoassay, immunohistochemistry, and flow cytometry are also used to diagnose a variety of cancers. Although many of the present technologies and processes are expensive, invasive, time-consuming, challenging, and limited to top-notch laboratory facilities, they have the potential to produce both accurate and inaccurate findings [8]. More specifically, there is fear that frequent use of these diagnostic procedures may result in cancer or other hazardous and permanent effects. For instance, Shao et al. discovered that exposure to medical radiation from CT scans in adults and children is linked to a higher risk of thyroid cancer and leukemia [9]. Pearce et al. found that some kids who underwent CT scans later had leukemia and brain tumor diagnoses because they are extremely sensitive to ionizing radiation [10]. Another study discovered a possible connection between CT angiography scans of the body parts and the occurrence of cancer in healthy persons [11].

Additionally, due to the possibility of tumor aggressiveness, the American Society of Clinical Oncology has advised physicians to avoid using advanced imaging diagnostic modalities (CT and PET) for patients with stage I and II BC [12]. Comparing cancer incidence rates in individuals who received a CT scan more than a year before receiving a cancer diagnosis to those in unaffected individuals showed that the possibility of cancer incidence in people exposed was 24% higher than in people who were not exposed [13]. As a result, additional strategies are required to diagnose BC with high accuracy and little danger [14].

A biosensor for cancer detection typically comprises an oncomarker as a target molecule, a bio-receptor as a recognition element, and a suitable bio-transducer with high specificity and affinity [15]. EN has several advantages such as simplicity and cheapness, mass production, and use as point-of-care testing (POCT) tools in doctors’ offices or patients’ homes, among many other biosensors [16]. These also have the advantage of quick response due to direct measurement in physiological fluids. According to these characteristics, research into creating ultrasensitive EN with high selectivity for detecting cancer oncomarkers has received significant attention in recent years [17]. The development, growth, and metastasis of tumors produce several unique cancer indicators, including altered genomic, circulating tumor DNA, circulating tumor cells, microRNA, cancer antigens, cytokines, matrix metalloproteinases, exosomes, and others [18,19]. The most common analytes utilized as oncomarkers are markers on the cell surface or their shed-off extracellular domains in the serum [20]. To recognize an oncomarker, some recognition elements known as bio-receptors, are used which interact with oncomarkers and cause a dose-dependent response.

Furthermore, signal enhancers are occasionally nanomaterials or chemicals with high adsorptive qualities in the system to accomplish the required signal amplification. This study gives an overview of current achievements in EN for blood-based oncomarker detection and analysis in various stages of BC. The review has concentrated on the many types of oncomarkers and detection methodologies based on the function of different nanomaterials and signal amplification schemes for BC detection.

## 2. Breast Tissue Pathobiology

In humans, the breast serves a variety of purposes. A distinguishing characteristic of animals is the mammary gland, primarily responsible for producing milk to feed babies. Before puberty, males and females have only a few ducts in their breasts. Progesterone and estrogen hormones during puberty cause the growth and development of women’s breasts [21]. The breast contains 15 to 20 milk ducts. Small channels allow breast milk to exit the body when the duct ends at the nipple [22]. The breast ducts are protected by myoepithelial cells, which are held in place by connective tissue stroma and varying amounts of fat [23]. The terminal duct lobular units enlarge during pregnancy. Oxytocin and prolactin release results in the production of milk [24].

**Figure 1:**
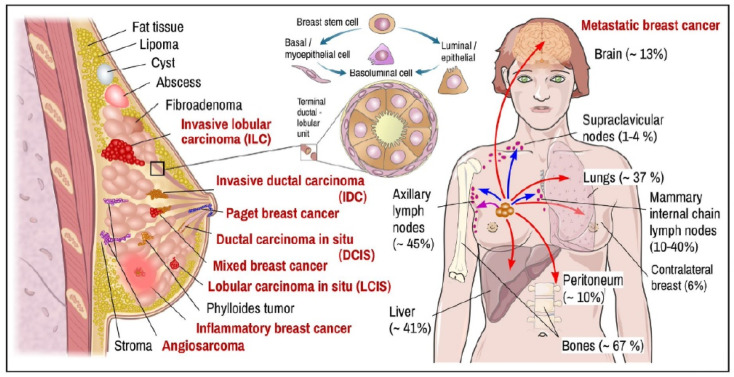
BC in women. Histological and molecular characterizations have significant effects on the treatment procedures. Left: the main histopathological BC types (red) and other important pathobiological findings. Middle: ductal-lobular terminal unit showing the location of basal-myoepithelial cells and luminal cells. Right: locations that are frequently involved in BC metastasis. Arrows indicate the locations of metastatic spread in the body: violet—local, blue—through lymph, and red—via blood. Reprinted by permission from Pubmed. NCBI, ref. [29], copyright 2022.

The lymphatic drainage of the breast is essential from a therapeutic perspective. About 5% of breast lymph drains to nodes along the internal mammary arteries through the intercostal spaces. One or two larger channels carry 95% of the lymph toward the axilla. Therefore, to check for lymph node involvement, auxiliary surgery should be performed on all patients with invasive BC [25]. BC is a complex illness with several subgroups and hereditary mutations. The mammary gland, fibrous and adipose tissue are among the anatomical structures of the breast gland where breast tumors can grow. The most frequent kind of malignant tumor is carcinoma. The terminal lobes and ducts of the mammary gland are the most common causes of BC (Figure 1). The ductal subtype accounts for over 80% of all diagnosed BC cases [26]. Of all identified cases, 40–75% fall within one of these two categories. Furthermore, 10% of cases also involve other cancers, such as inflammatory BC, male BC, breast Paget’s disease, papillary carcinoma, and others [27,28].

BC is categorized based on histology into four types: ductal carcinoma in situ (DCIS), lobular carcinoma in situ (LCIS), invasive ductal carcinoma (IDC), and invasive lobular carcinoma (ILC) [30]. DCIS is pre-invasive cancer that occurs when aberrant cells are detected in the lining of the breast milk duct. DCIS is a very early stage of cancer that is often treatable but can potentially spread to the breast tissue surrounding it if ignored or undetected. DCIS is a cancer that has not spread beyond the milk duct and is present there [31,32]. About 1 in 5 new BCs will be DCIS [33]. Almost all women with BC at this early stage are curable. Stage 0 is the earliest stage of BC (carcinoma in situ). Stages I through IV are then included. Stage 0 BC or intraductal carcinoma are additional names for DCIS. A higher number generally means more cancer spread [34]. 

LCIS is a malignancy in which abnormal cells are detected in the breast lobules. LCIS is largely treated and rarely progresses to invasive carcinoma. LCIS in one breast, on the other hand, increases the chance of getting BC in either breast [35]. The distinction between LCIS (or lobular neoplasia) and DCIS is that LCIS refers to lobular cancer, whereas DCIS refers to milk duct cancer. Stage 0 BC includes noninvasive BC, DCIS, and LCIS [36]. 

The most common type of BC is invasive ductal carcinoma (IDC), an aggressive type of cancer in that abnormal cancer cells expand from the milk ducts to another breast tissue. IDC, which affects the ducts responsible for transporting milk through the breast, includes around 8 out of 10 BC diagnoses [37]. IDC begins in the breast milk duct’s lining cells. Then, cancer spreads into the nearby breast tissues after penetrating the duct wall. It can reach other body parts at this stage through the lymphatic system and blood flow [38]. When treated early on, localized IDC has a remarkable five-year survival rate that approaches 100%. According to the size of the tumor and the extent of its spread, IDC can be classified as being in stages I (the earliest stage) through IV (the most advanced stage). Stage IV describes malignancies that have migrated from the breast to other body areas, such as the bones or liver, whereas stages I, II, and III represent early-stage tumors [39].

Infiltrating lobular carcinoma (ILC) is a type of BC that begins in the breast’s lobules (milk-producing glands) and extends to surrounding normal tissue. As an invasive type of cancer, ILC spreads beyond its original tumor site. Over time, ILC may become metastatic BC. ILC is slow-growing compared to other BCs. As it is difficult to detect on a mammogram, these tumors can be large by the time they are diagnosed [40]. When diagnosed and treated early, ILC has a remarkable five-year survival rate compared to other cancers—nearly 100%. After IDC, ILC is the second most typical kind of BC. ILCs makeup 1 in 10 invasive BCs and can appear at any age, although older persons seem to have it more frequently [41]. After menopause, hormone replacement therapy may increase the incidence of BC, according to some research [42].

ILC does not usually appear well on a mammogram; thus, an MRI may be required. The distinction between ILC and LCIS is significant. LCIS suggests the cancer is still in the milk glands and has not spread to other areas. More than 80% of patients with ILC possess estrogen receptor (ER) positive and Human Epidermal growth factor Receptor-2 (HER2) negative. ILC can occasionally become larger than it does in mammography because of the way it develops. It is frequently identified as a more advanced stage of cancer. If BC spreads to other organs, it often affects the colon, uterus, ovary, stomach, lung, bone, and others [43].

Based on immunohistochemistry analysis of the key proteins, BC can be divided into four subgroups: ER, progesterone receptor (PR), Hormone Receptor (HR), HER2 proto-oncogene, and proliferation Ki-67 antigen [44]. This method is the most commonly accepted classification of BC from the point of view of immunohistochemistry. BC is divided into four main categories according to whether tumor-expressing factors such as HR and HER2 are present or absent: luminal A (HR+/HER2-), luminal B (HR+/HER2+), HER2 positive (HR-/HER2+), and triple-negative (HR-/HER2-) [45]. There are few treatment options for triple-negative BC (TNBC), as it resists endocrine and particularly targeted therapies. It is aggressive and has a high recurrence rate and poor prognosis [46]. Endocrine therapy is a medication that blocks estrogen’s impact on BC cells. Basal-like BC (BLBC) accounts for 10–15% of all BCs and has an expression profile resembling the normal breast myoepithelial cell compartment. BLBC has the lowest clinical outcome of any BC subgroup. This subtype of tumor lacks ER, PR, and HER2 receptors while expressing significant immunohistochemistry oncomarkers such as cytokeratins 5, 14, and 17. BLBCs typically comprise 60–90% of TNBC and are characterized by a fast-moving disease course, earlier age of occurrence, and a lack of targeted treatment [47]. The main features of TNBC, like BLBC, include that it affects younger people less than 50 years old, has a high prevalence in African-American women, and is more aggressive than tumors derived from other molecular subtypes. In contrast, BLBC cells have different protein changes than TNBCs [48].

Neoadjuvant endocrine therapy is increasingly employed to treat BC depending on the presence of oncomarkers, including ER, PR, and Ki-67. A nuclear protein called Ki-67 antigen, coupled with genes that promote tumor proliferation, represents cell proliferation and is strongly related to BC differentiation and tumor metastasis. The proliferative activity measured by Ki-67 represents cancer’s aggressiveness, treatment response, and time of cancer recurrence [49]. The high expression of Ki-67 also reflects low survival rates. BLBCs are divided into the luminal A and B groups using the Ki-67 expression [50].

The incidence, medication response, and illness progression vary between each subgroup. About 15% of BC patients belong to the triple-negative category, which is devoid of both HR and HER2 expression, whereas about 70% of BC patients belong to the luminal A or B subgroup. Furthermore, 20% of BCs have HER2-amplified cells with poor prognoses [51]. HER2+ cancers can be treated with monoclonal antibodies such as pertuzumab, trastuzumab, and/or tyrosine kinase inhibitor lapatinib, and HR+ tumors can be treated with anastrozole, letrozole, tamoxifen, or exemestane [52,53]. The most aggressive subgroup of BCs, with the highest risk of relapse within five years, is the triple-negative subgroup [54]. It should be noted that mutations significantly influence BC development and progression in tumor-suppressor genes like TP53, BRCA1 and 2, and PTEN. The response to chemical, hormonal and molecular treatments depends mainly on clinical results at diagnosis, the occurrence of genetic mutations, and specific subgroups [55].

## 3. Blood-Based Oncomarkers of BC

Blood tests for cancer detection are more practical, noninvasive, widely recognized, repeatable, and economical than imaging and biopsy techniques. Specialized proteins, nucleic acids, or other cellular vesicles are routinely produced by cancer cells, and these cells can also secrete living or dead cells into the blood. Examining blood for the presence of such components may offer a method for detecting cancer. Various blood-based oncomarkers with the potential for clinical application to early detection of BC have been discovered due to advancements in genomics, proteomics, and glycomics [56]. Since blood aspiration is just a mildly intrusive operation, serum oncomarkers are crucial for monitoring cancer patients and giving vital information about the illness. As oncomarkers are available in the blood and convey information about the health situation, they have considerable potential for screening and diagnosis [57]. 

### 3.1. Proteins of Carcinoma in the Circulation

The proliferation, invasion, metastasis, aggressiveness, angiogenesis, oncogenic signaling, and immune control of tumor cells have all been linked to cancer proteins. For this reason, they may work as oncomarkers for cancer detection [58]. Serum carcinoma oncomarkers in BC include carcinoembryonic antigen (CEA), carbohydrate antigen (CA) 125 (CA-125), CA15-3, CA27-29, HER2, epidermal growth factor receptor (EGFR), a cluster of differentiation 44 (CD 44), epithelial cell adhesion molecule (EpCAM, CD326), Ki-67, tissue polypeptide-specific and tissue polypeptide antigen [59]. None of these oncomarkers is currently used alone and exclusively for screening because their specificity and diagnostic sensitivity are restricted to identifying the early stages of the disease. These oncomarkers are primarily used to evaluate treatment response in patients with advanced BC.

### 3.2. Circulating Tumor Cells (CTC)

The peripheral blood of cancer patients may become contaminated with tumor cells through passive or active intravascular shedding from primary or metastatic tumors. When CTCs are present in the early stages, the probability of recurrence and death increases [60]. CTC measurements for BC diagnosis should have high sensitivity and reproducibility. The number of CTCs in the bloodstream is limited, with only one CTC per billion normal blood cells [61]. Therefore, more sensitive and specific technologies are needed to track and identify a wide and diverse range of CTCs in the blood.

### 3.3. Circulating Tumor-Specific DNA (ctDNA)

Bloodstream ctDNA has become a potential indicator of disease status in BC [62,63]. Increased ctDNA levels are associated with advanced stages of BC and metastases [64]. BC patients have significantly higher ctDNA levels than healthy controls [65]. Recently, ctDNA detection and quantification as a serum oncomarker for noninvasive diagnostics, therapy response monitoring, risk stratification, and recurrent disease detection has grown in importance [66]. ctDNA is also found in most patients with metastatic BC. It has also been found that ctDNA mutation analysis can detect early-stage cancers [67].

### 3.4. Circulating miRNAs

The microRNAs are non-coding RNA tiny molecules containing about 22 nucleotides. Its role in the RNA molecule involves post-transcriptional control of gene expression and silencing. Base pairing to complementary sequences in the 30 untranslated regions of target mRNAs allows these regulatory RNA molecules to regulate the production of the target mRNAs. When compared to healthy individuals, a small number of miRNAs were discovered to be downregulated in BC, while a large number of miRNAs were revealed to be considerably elevated in the blood of patients. Oncomirs target tumor-suppressor genes and activate oncogenic transcription factors to carry out their oncogenic action [68].

### 3.5. Extracellular Vesicles (Evs)

All types of vesicles in the extracellular space are collectively called “Evs”. They are exosomes, microvesicles, microparticles, ectosomes, oncosomes, prostasomes, tolerosomes, and apoptotic bodies [69]. All cells, including cancer cells, release Evs for intercellular transportation and communication. They can be identified in liquid biopsies in all body fluids. Tumor-derived Evs are potential novel oncomarkers for early detection, prognostication, and cancer surveillance due to their presence in circulating body fluids. Evs are a diverse group of materials transferred from parental to target cells that alter phenotypes in the tumor microenvironment. These molecules include oncoproteins, oncopeptides, DNA fragments, RNA species, and lipids. According to data, Evs play crucial roles in early and late processes connected to tumor development and metastasis, depending on their particular cargo and potential role in the early detection, monitoring, and progression of cancer, response to chemotherapeutic treatment, and development of novel targeted therapeutics. Tumor-derived Evs have been found in circulating body fluids and are known as novel oncomarkers in BC [70].

## 4. Electrochemical Biosensors Based on Nanostructured Materials as Ultrasensitive Platforms for Detecting BC Oncomarkers

Nanotechnology is a promising technology for biosensor applications in various ways. The biosensors need to have high selectivity and stability characteristics with practical significance. One of the most prevalent and economically successful classes of biosensors is the electrochemical biosensor (EB) family. Biosensor sensitivity, the limit of detection (LOD), stability, reaction time, linearity, and other analytical properties are all greatly enhanced by using nanomaterials. New functional nanomaterials (NMs) are crucial parts of several biosensors. Nanostructured metal nanoparticles (NPs), semiconductors, carbon materials, and polymer NPs are finding increased applications in developing nano biosensor devices [71]. NMs, depending on the transduction mechanism, can be exploited for enhanced immobilization of biomolecules, electrical conduction, amplification of the signal, and detection of nanoprobes and electroactive species. These biosensors have been categorized and reviewed based on applying NMs, analytes, different diagnostic techniques, and suitability for use with real samples.

### 4.1. Carcinoma Proteins in the Circulation

Protein molecular oncomarkers have a very high level of popularity, as evidenced by the availability of a wide variety of bioanalytical tools that can measure and identify proteins in complex biological fluids [72].

#### 4.1.1. CA15-3

The normal range of CA 15-3 should equal or less than 30 units per milliliter (U/mL). Higher CA 15-3 levels are associated with advanced stages of BC and a larger tumor burden. If the tumor produces CA 15-3, the levels of oncomarker will increase as the tumor grows. The highest levels of CA 15-3 oncoprotein may exist in metastatic BC. Especially when metastases occur to the bone or liver [73].

Kuntamung describes an electrochemical immunosensor (EI) to detect CA15-3 BC oncomarker. Gold NPs coated with redox species-antibody-conjugated polyethyleneimine (PEI) are deposited to create the immunosensor. The immunosensor can determine the oncomarker with a reduction of oxidation peak current in a single run measuring. The current is impressively lower than the clinically relevant level [74]. 

To measure CA 15-3, Rebelo et al. created an EB. This chip, including gold screen-printed electrodes, was utilized to create a portable device for keeping an eye on the oncomarker in a POCT for a medical setting. Low detection levels (LOD: 0.95 U/mL) and a broad linear concentration range (1.0 to 1000 U/mL) were both accomplished by the sophisticated biosensor [75]. Martin and his colleagues created a sandwich-type EI with a low LOD of 0.08 fg/mL. A layer-by-layer sheet of reduced graphene oxide and gold NPs served as the immunosensor matrix on screen-printed carbon electrodes. High sensitivity is achieved by using secondary antibodies labeled with horseradish peroxidase in the presence of hydrogen peroxide (HRP) as signal amplifiers and hydroquinone as an electron mediator [76].

Oliveira et al. fabricated an electrode using a paper layer containing carbon nanotube (CNT), AgNP, and graphite pencil. AuNPs and a molecularly imprinted polymer (MIP) were assembled on the electrode. The sensor was used in serum saliva samples’ CA 15-3 determination. However, its application in saliva was not satisfactory [77].

A novel label-free EI for detecting CA 15-3 was developed using ternary silver/titanium dioxide/rGO nanocomposites as a basis for signal enhancement. A potential signal amplification mechanism of the amperometric detection was investigated by tracking the decrease in HRP and investigating the electrocatalytic current response via the immunoreaction between CA 15-3 antibody–antigen on the surface of the developed nanocomposite immunosensor. In human blood samples with an extremely low LOD, a linear concentration of 0.1–300 U/mL was achieved (0.07 U/mL) [78].

A solvothermal process using l-carnosine as the co-structure-directing agent was developed to manufacture PtCo alloyed nano dendrites (PtCo NDs). The PtCo NDs provided many active sites for the electrocatalytic oxygen reduction process. This resulted in creating an EI for the extremely sensitive CA15-3 test. The biosensor had a broad linear range of 0.1–200 U/mL and a low LOD of 0.0114 U/mL, permitting it to be used successfully with diluted human blood samples [79]. The creation of a bio-receptive interface for recognizing a BC oncomarker CA15-3 was described by Gajdosova et al. Mxene was used to form the conductive interface, and a diazonium moiety was used to electrochemically graft a mixed layer of sulfo betaine and carboxy betaine onto that material. Using a modified interface, the anti-CA15-3 antibody was then covalently immobilized as a probe for CA15-3 detection. In the ultimate construction of an immunosensor, two distinct strategies were applied: an interface that was eventually closed by bovine serum albumin (BSA) or an immunosensor without that alteration. A soluble redox probe, Ru(NH_3_)_6_^3+^ ion, was used in EB reading to detect CA15-3 [80].

#### 4.1.2. Carbohydrate Antigen 125 (CA 125)

CA 125 in the range of 0 to 35 U/mL is considered normal. An increase in the level of this antigen to more than 35 U/mL can indicate the presence of cancer or other diseases [81].

A biosensor was created to detect CA-125 BC oncomarker using the EB technique in a study by Er et al. Electrophilic cyclization procedures and Pd-catalyzed coupling reactions provided a novel benzothiophene derivative known as 5-(2-phenylbenzo[b]thiophen-3-yl) thiophene-2-carbaldehyde (PTTC). After evenly dissolving PTTC in Nafion solution, ink was generated. Before incubating CA-125, the glassy carbon electrode (GCE) was covered with this ink. The PTTC-based GCE made under ideal circumstances had a 1–100 ng/mL range, as determined by differential pulse voltammetry (DPV). LOD values were determined to be 0.0096 and 0.0288 ng/mL [82].

To detect CA-125, novel label-free EI with boron nitride nanosheet modifications were created. The surface of the screen-printed electrode (SPE) was altered by applying boron nitride nanosheets. Since the anti-CA-125 antibody naturally has an affinity for boron nitride nanosheets, it was immediately adsorbed onto the electrode surface. The newly synthesized immunosensor displayed acceptable detection characteristics for CA-125. The linear range for the CA-125 oncomarker was 5–100 U, and the LOD was 1.18 U/mL. This immunosensor successfully detected CA-125 in artificial human serum samples [83].

**Figure 2:**
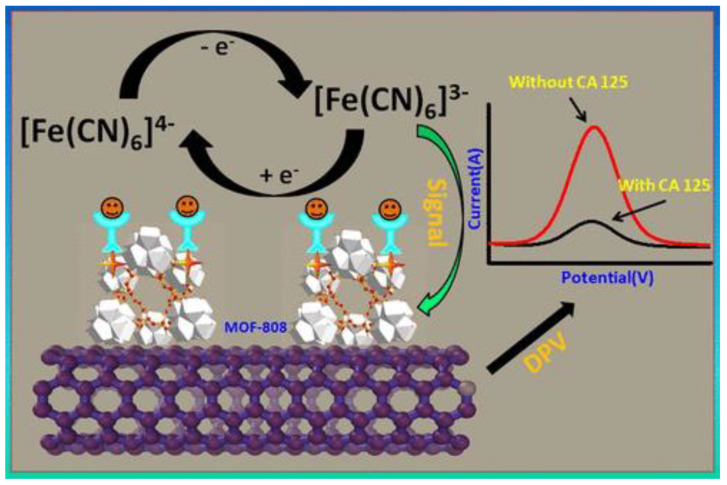
Illustration of the label-free electrochemical immunosensor fabrication. A glassy carbon electrode modified with MOF-808/CNT composite was used as a platform. The antibody binding sites of MOF-808/CNT were enriched by functionalization with streptavidin. Adapted with approval from [84]. Copyright 2015 American Chemical Society.

A nanocomposite of CNT and Zr-trimesic acid metal–organic framework (MOF) [MOF-808] was generated using the in situ synthesis of MOF-808 on activated CNT. The properties of MOF-808, large surface area, and electrocatalytic behavior were applied to create an immunosensor for detecting CA-125 oncomarker at extremely low levels. The MOF-808/CNT composite components collectively gave significantly better EB properties. A GCE customized with MOF-808/CNT was used as a platform to construct a label-free EI (Figure 2). This immunosensor had an LOD of 0.5 pg/mL, ranging from 0.001–0.1 to 0.1–30 ng/mL, with great selectivity and reproducibility [84]. 

CA-125 identification is critical for diagnosing and treating BC. A biosensor was developed to detect the CA-125 oncomarker in human plasma samples. The anti-CA-125 antibody (Ab) was bound on a matrix containing AgNPs put on conductive nano-ink modified CysA-Au NPs with D-penicillamine-functionalized graphene quantum dots. The immunosensor performed well and had a high level of sensitivity for detecting CA-125 oncomarker [85]. The peptides and aptamers were co-immobilized in one step onto the electrode surface that had been treated with electrodeposited poly (3,4-ethylene dioxythiophene) (PEDOT) and AuNPs to create the electrochemical aptasensor. The developed peptide-based aptasensor displayed a reasonable response for CA-125 measurement, with a very broad linear range (0.1–1000 U/mL) and a modest LOD (0.027 U/mL) to detect CA-125 in clinical samples with an acceptable degree of accuracy [86].

Li et al. developed an EB using tri copper benzene-1,3,5-tricarboxylate (CuBTC) and molybdenum disulfide (MoS_2_) created through a hydrothermal process to improve the capacity of electron and ion transfer. To make better the bond between CA-125 antibodies and the substrate material, AuNPs were electro-reduced on a CuBTC@MoS_2_-modified electrode. CuBTC@MoS_2_ and AuNPs worked in concert to produce biosensors that performed well electrochemically. CuBTC@MoS_2_-AuNPs/CA-125 Ab-functionalized electrodes were employed to detect the BC oncomarker CA-125 with LOD of 0.5 mU/mL at concentrations between 0.5 and 500 U/mL [87].

A universal approach was provided for creating extraordinarily sensitive biosensors based on antifouling peptides and recognizing DNA probes. The peptide was terminated with biotin and azide groups at the bottom to make the peptide-DNA conjugate. Then, it was coupled with a common DNA aptamer modified with 5′-dibenzocyclooctyne. The EB was built using streptavidin (SA)-assisted immobilization of straight peptides and peptide-DNA conjugates onto an electrode surface modified with electrodeposited PEDOT and AuNPs. The constructed biosensor demonstrated remarkable antifouling in body fluids [88].

A smartphone-based device with DPV measurement was developed to detect CA-125. The system comprised a screen-printed immunosensor, a tiny EB detector, and an Android smartphone. The information was obtained via Bluetooth on an Android smartphone. The detector was powered by a 3.3 V button battery, USB, and an audio connector on the smartphone. The results of these studies were shown on a smartphone through an Android application. The experimental results demonstrated a strong linear relationship between DPV peak currents and the logarithm of CA-125 concentrations ranging from 1.3 to 260 U/mL with an LOD of 2 mU/mL [89].

An aptasensor based on magnetic Fe_2_O_3_/Fe_3_O_4_ heterogeneous hollow nanorods was developed to detect CA-125. The hydrolysis–calcination technique was successfully used to make magnetic Fe_2_O_3_/Fe_3_O_4_-Au nanocomposites. The DNA aptamer probe was connected to nanocomposites to boost the current signal [90].

A voltammetric biosensor of nitrogen-doped rGO (NrGO) nanocomposites, thionine (Thi), and AuNPs detects CA-125. The variation in DPV production is examined using carbon nanofiber (NrGO/CNF) and carbon nanotube composites (NrGO/CNF). Using carbon variations increased the signal amplification and sensitivity of the produced sensor. Furthermore, it provided a wide range of current output, indicating the possibility for detection in a range of concentrations (up to 32 × 10^−4^ U/mL). LOD of 0.28 U/mL resulted in higher NrGO/CNF sensitivity. Consequently, an immunosensor for commercial and clinical antigens was developed and tested utilizing dual antibodies [91].

#### 4.1.3. CA 27-29

In general, the higher the antigen level of CA 27-29 (>38 U/mL), the more advanced BC or larger the tumor burden. As the tumor grows, the level of CA 27-29 increases. The highest level may be seen in the case of metastasis to the bone or liver [92].

Patients who receive clinical treatments for BC and an early diagnosis have a great chance of surviving. The CA 27-29 antigen was identified using a label-free ultrasensitive EI by Au/MoS_2_/rGO nanocomposite technology. The significant synergistic combination of AuNPs, MoS_2_, and rGO resulted in the nanocomposite’s exceptional EB sensitivity. The amperometric detection signal was amplified by measuring the electrocatalytic current response of HRP reduction via the immunoreaction between the anti-CA 27-29 and CA 27-29 antigens on the surface of the nanocomposites. The immunosensor showed great sensitivity toward recognizing CA 27-29 BC antigen in human serum with an LOD of 0.08 U/mL [93].

#### 4.1.4. CD44

Extensive studies have shown that CD44 is widely overexpressed in various cancer types, such as pancreatic cancer, TNBC, lung cancer, and prostate cancer. 

Extensive studies show that CD44 is widely expressed in various cancers, including prostate, TNBC, lung, and pancreatic cancer. CD44 is one of the most widely used oncomarkers for identifying cancer stem cells (CSC). CSC in BC are the source of cells resistant to chemotherapeutic agents in different solid tumors and are responsible for recurrence and metastasis. Moreover, these cells have high self-renewal and differentiation capacity [94,95,96,97].

A novel graphene quantum dot (GQD)-based EB has been developed for the rapid and extremely accurate primary identification of the BC oncomarker cluster of CD44 antigen. The EB responds to CD44 antigen concentrations in blood samples from 1.0 pg/mL to 100.0 ng/mL, with an LOD of 2.71 fg/mL. According to the findings, the developed GQDs can be used as a viable nanomaterial in biosensing applications [98].

Protein MIP with ultra-high specificity that is easy to make, chemically stable, and simple to combine with a transducer provides high-performance for a wide variety of targets. A dual-responsive EB was presented that combines synthetic protein MIP and natural hyaluronic acid (HA) probes into a flexible electrode to detect CD44 oncomarker. Screen-printed electrodes (SPEs) with a dual-channel design were developed. Protein MIPs were produced as a single responsive channel by polymerizing the template protein CD44, a biocompatible antifouling agent, and a functional alginate gel monomer. To create the other responsive channel, natural HA probes were made immobile. MIPs and HA are more specific, antifouling, and biocompatible. Different batch integrations of functionalized materials and active biomolecules into EB and electrocatalytic biosensors might be created for diverse components to monitor physical indications and diagnose people’s ailments as part of the dual-responsive technique [99].

Biomolecule passive adsorption and interference in Ebs are of great interest. In a study, a sensitive sandwich-type antifouling immunoassay was developed using platelet membrane/AuNP/delaminated V2C nanosheet-modified electrode serving as the biosensing interface substrate and methylene blue(MB)/aminated MOF serving as an EB signal probe. For measuring CD44, the suggested antifouling biosensor offers a good analytical performance [100].

Chen et al. established a very sensitive EI to identify the BC oncomarker CD44 antigen. A hybrid nanocomposite was made by AuNPs immobilized on a GCE, graphene oxide (GO), and ionic liquid (IL). GO enhanced antibody immobilization due to the accessibility of oxygen functionalities. Adding AuNPs and 1-butyl-3-methylimidazolium tetrafluoroborate boosted electron transport and improved immunosensor effectiveness. DPV and electrochemical impedance spectroscopy (EIS) methods were used to identify CD44 antigens quantitatively. Conjugating HA and BSA-modified AuNPs to assess CD44 expression in cancer cells resulted in a new label-free EB based on EIS. The EIS measured the change in electron transfer resistance (R_et_) to count cancer cells and quantify the degree of CD44 expression on the cell surface [101].

#### 4.1.5. CEA

Since CEA is now recognized as a broad-spectrum cancer oncomarker, precise sample measurement can benefit early detection and therapy. Positive values of CEA are those above 5 µg/L. Exonuclease III and a hybrid chain reaction were used to construct an electrochemical aptasensor for CEA detection. Due to the high interaction between CEA and the aptamer area of hairpin probe 1, Due to high interaction between the target CEA and the aptamer area of hairpin probe 1, a considerable amount of hairpin probe 1 and 2 (H1 and H2) double chain compound was synthesized by H1 the residual H1 triggering catalytic hairpin assembly created a considerable amount of H1 (referred to as H1) and hairpin probe 2 (referred to as H2) double chain compound (referred to as H1@H2). Exonuclease III disassembled the H1@H2 pair, releasing H1 to trigger the initial signal amplification. A hybrid chain reaction initiated by numerous designed trigger chains resulted in a second signal enhancement. A wide linear range of 10 pg/mL to 100 ng/mL and a low LOD of 0.84 pg/mL of this biosensor demonstrated remarkable analytical performance for detecting CEA. Furthermore, this biosensing approach verified the accurate determination of CEA in serum samples [102].

CEA is commonly used in the clinical diagnosis of various tumor forms. To assess the CEA oncomarker, an EI was described. Electrospinning at the surface of the discharged battery coal electrode produced polyacrylonitrile nanofibers (PANnf) packed with titanium (IV) oxide NPs (TiO_2_NPs) and CEA antibodies (Anti-CEA) as the CEA oncomarker receptor (DBC). The third step was identifying CEA using DBC/PANnf + TiO_2_NPs/Anti-CEA. This hybrid device was a cutting-edge immunosensor development technique for label-free CEA detection. According to the findings, the device could detect even minute variations in the CEA concentration [103]. The first electrically facilitated atom transfer radical polymerization (eATRP) and Polyethylenimine (PEI)-based EB was developed with dual signal amplification for very sensitive CEA detection. Compared to others, this EB used a less complicated and more ecologically friendly eATRP, and the electron transfer rate increased by PEI [104].

A high-performance sensing layer based on dual-template MIP was developed to efficiently detect CEA and alpha-fetoprotein (AFP) oncomarkers. Plastic antibodies against AFP and CEA were created utilizing an electropolymerized polypyrrole electrode doped with fluorine. Since methyl orange improved the conductivity of polypyrrole, rectangular polypyrrole polypyrrole-doped methyl orange nanotubes were formed. The LOD for CEA and AFP were 1.6 and 3.3 pg/mL. The dual-template MIP sensor’s high sensitivity and improved stability made it a viable technique for identifying AFP and CEA in serum samples [105].

fA CEA oncomarker EB was developed using human breast ductal adenocarcinoma (MCF-7) and human ovarian adenocarcinoma cancer cells. Chitosan (Cs) matrix dispersed with MoS_2_ nanostructures and AuNP improved the graphite electrodes used in disposable pencils. The surface of the antibody and electrode interacted electrochemically more easily. The immunosensor demonstrated remarkable sensitivity to CEA oncomarker under ideal circumstances, with an LOD of 1.93 ng/mL. MCF-7 cells that were CEA-positive exhibited superior adhesion and attachment capabilities to surfaces coated with MoS_2_/Cs/Au/Anti-CEA/CEA [106]. To detect a CEA oncomarker, Ranjan et al. created an EI based on zirconia (ZrO_2_)-rGO-1-ethyl-3-methylimidazolium tetrafluoroborate IL nanocomposite. The produced nanocomposite had a larger active surface area, strong oxygen functionality, and great electroconductivity, which enhanced high anti-CEA antibody loading [107].

A sandwich-type electrochemical aptasensor was designed to assess two important oncomarkers in BC, CEA, and CA 15-3. An AuNP three-dimensional graphene hydrogel (AuNPs/3DGH) nanocomposite was used as a biosensing substrate. AuNPs, redox probes, and graphene nanocomposite were combined with CEA and CA 15-3 aptamers as biosensing probes. Hemin and ferrocene were used as redox probes for CEA and CA 15-3 and as EB signals to detect dual oncomarkers. To immobilize the surface of an AuNPs/3DGH-modified electrode, the CEA and CA 15-3 aptamers were utilized. Peak currents and potentials in DPVs correlated with oncomarker concentration and characteristics. The results agreed with the ELISA approach, demonstrating the high reliability of the aptasensor [108]. Using polypyrrole nanocomposite film electrodes, researchers developed a foldable free-standing EB to detect CEA. The sandwiched structure of Ppy doped independently with pentaerythritol ethoxylate and 2-naphthalene sulfonate was used to create the conducting Ppy composite. To build a free-standing EB, AuNPs were electrodeposited onto a Ppy composite sheet and self-assembled to a CEA aptamer. This Ppy composite film-based EB performed well for CEA detection [109].

A click reaction was effectively used to create an EI capable of detecting human blood targets with little fouling and great sensitivity. Using the streptavidin-biotin affinity interaction, one planned biotinylated peptide was associated with another peptide containing biotin and azide groups, which was then bound to the electrode to create an antifouling interface. The exposed azide group on the branching peptide was then coupled to an antibody strengthened with 5′-dibenzocyclooctyne, and the click reaction between the azide group and dibenzocyclooctyne group was recorded by the immunosensor. In this work, antifouling peptides were effectively coupled with different antibodies to construct a range of biosensors [110]. Rare earth metals can form stable metal–organic frameworks (MOFs). However, this promise had not been completely realized. The solvothermal method was used to create samarium (Sm)-based MOFs with three distinct chemical linkers—trimesic acid, meso-tetra(4-carboxyphenyl) porphine, and 1,3,6,8-tetra(4-carboxyphenyl) pyrene. The produced MOFs were utilized to produce immunosensors that recognized CEA utilizing a label-free signaling method after their EB characteristics were investigated. The repeatability and selectivity of immunosensor were within acceptable ranges [111].

The single tumor antigen lacks the sensitivity and selectivity needed for precise diagnostic criteria, and its measurement is susceptible to inaccurate positive and negative interpretations. Yang et al. used a variety of signal amplification methodologies to construct an EB that recognized two cancer markers, cytokeratin 19 fragments 21-1 (CYFRA21-1) and CEA. Many AuNPs are scattered on the surface of poly-thionine, three-dimensional graphene, and poly-m-cresol purple, providing numerous antigen–antibody binding sites and improving the EB signals [112].

Polyoctopamine is a non-conducting polymer with amine functionalization that allows flexible covalent coupling via thiol linker conjugation and carboxyl or aldehyde functional groups without requiring pre- or post-surface activation. The cancer marker CEA was employed as the target analyte, and an antibody and a synthetic binding protein were used as distinct bio-receptors to demonstrate the application of poly octopamine as a transducer polymer layer for guided immobilization of bio-receptors. Human serum infusion was used to assess the effectiveness of newly constructed POCT-based biosensors. The results demonstrated that octopamine electropolymerization on screen-printed gold electrodes (SPGEs) produced a thin, low-resistance polymer sheet. The proximity of the immobilized bio-receptors to the transducer layer significantly improved sensitivity. The LOD of the smaller monomeric bio-receptor was 11.76 fM, significantly lower than the clinically relevant baseline values of 25 pM, which was similar to the sensitivity to detect CEA of the dimeric antibody [113].

A CEA oncomarker detecting EB was Integrated into a direct methanol fuel cell (DMFC) electrical circuit, which ran in passive mode and functioned as both a power source and a signal transducer. A fluorine-doped tin oxide (FTO) conductive glass substrate was coupled to the DMFC negative pole side through a molecularly imprinted polypyrrole and a conductive layer as the CEA sensing layer. The sensing element was then covered with a cover FTO glass connected to the positive side of the DMFC and secured with a clip. Due to the obvious MIP layer interfaced in the electrical circuit and the high stability of signals, the power curves of the DMFC/Sensor integrated system indicated lower power values when compared to control DMFCs [114].

#### 4.1.6. HER2

HER2-positive cells are those with higher than normal levels of HER2 (>15 ng/mL) [115]. The status of HER2 oncomarker is an important component of early-stage BC screening, diagnosis, and follow-up. The creation of EB based on aptamers is essential for quantitative and qualitative analysis of HER2 oncomarker. 

The nanocomposite employed in our investigation contained rhodium NPs on the graphite electrode surface and rGO nanosheets. Analytical and EB approaches were used to thoroughly explore nanomaterial fabrication and biosensor setup [116]. Creating an antifouling sensing interface based on the conducting polymer PEDOT and a biocompatible peptide hydrogel is a straight forward solution for the EB detection of HER2. The proposed short peptide Phe-Glu-Lys-Phe was employed to form a peptide hydrogel with the fluorene methoxycarbonyl group, allowing for good activity maintenance of the connected biomolecules. The PEDOT film also supplied a highly stable and conductible substrate. The peptide hydrogel-based biosensor has also demonstrated significant clinical application promise by being adaptable to complex biological specimens and detecting HER2 in serum samples with clinically acceptable accuracy [117].

Due to biocompatibility, environmental friendliness, and suitability of biopolymer films for in vivo studies, they are growing in demand for application in the POC industry. Their low conductivity, on the other hand, limits their capacity to deliver a reliable diagnosis. Nasrollahpour et al. created an enzyme-free EB based on electro-synthesized biocompatible WO_3_/poly glutamic acid nano-biocomposites to solve specific obstacles in the detection of circulating proteins in clinical samples [118].

To deal with the drawbacks of bare laser-scribed graphene (LSG) electrodes in terms of sensitivity, simple immobilization of detecting probes for biosensor manufacture, and simplicity of interface with POC devices, advancements in LSG-based electrodes are necessary. A class of LSG-AuNP EC sensing devices was introduced in a study, including LSG-AuNP working electrodes, LSG reference, and counter electrodes. LSG-AuNP electrodes were made by electroplating gold chloride solution onto LSG electrodes, demonstrating a 2-fold increase in electrocatalytic activity compared to ordinary LSG electrodes available on the market SPGEs. The LSG-AuNP aptasensor detected varying levels of HER2 in undiluted human serum [119].

A technique for EB detection of the BC oncomarker HER2 was provided in a study. The results of two techniques, successive adsorption, and electropolymerization, were examined to alter a GCE. The highly conductive polypyrrole was employed in the presence of sulfur/nitrogen-doped graphene quantum dots and a known cobalt phthalocyanine (CoPc). The HER2-specific HB5 aptamer was immobilized using an amide linkage on the various nanomaterials. When ferricyanide was utilized as the EB probe, the immobilized aptamer preferentially identified HER2 on the electrode interface, increasing the electrode charge transfer resistance. The immunosensors were extremely sensitive, with a best LOD of 0.00141 ng/mL. The results showed that the method was simple and sensitive enough to identify HER2 in blood samples with excellent reproducibility and accuracy [120]. Metallic nanostructures are considered to have the potential for developing new biosensors. Palladium (Pd) nanostructures constructed from oxidized carbon nanotubes enable label-free EB immunosensing of HER2. EB studies indicated a connection between the EB process and the nanostructured sensor, demonstrating the use of hierarchical Pd nanostructures supported on carbon nanotubes for HER2 detection. The enormous surface area of hierarchical Pd nanostructures allowed for an ultrasensitive response to HER2 with a detection range of 10 to 100 ng/mL. Pd nanostructuring has significant potential for chip-level POC screening of HER2-positive BC patients due to the EB response’s simplicity, sensitivity, and consistency in plasma samples [121]. 

The high fatality rate in British Columbia is due to misdiagnosis. HER2 is overexpressed in 20–30% of breast cancer tumors. The goals of this project include creating an EB for HER2 based on an AuNP-aptamer bioconjugate and investigating how DNA aptamer and HER2 interact using computational approaches. As a linker, polyethylene glycol, and maleimide were used to create the bioconjugate. The cysteamine-NH_2_ group changed the gold electrode, causing it to connect to the bioconjugate maleimide and establish a covalent link. The interaction of the bioconjugated aptamer with HER2 was studied using the [Fe (CN)_6_]^3/4^ redox system [122].

Applying rGO-polydopamine-grafted ferrocene(Fc)/Au@Ag nano shuttles (Au@Ag NSs) as electrode material and hollow Ni@PtNi yolk–shell nanocages-thionine (Ni@PtNi HNCs) as signal tags, Wang et al. developed a simple ratiometric EI for sensitively monitoring HER2 as a model analyte. The Au@Ag NSs significantly boosted the rGO-PDA-biocompatibility Fc while significantly boosting the Fc signals at the detecting interface. Furthermore, by incorporating the hollow structure, increasing specific surface area, and improving catalytic activity, the Ni@PtNi HNCs-Thi demonstrated a considerable improvement in Thi sensing signals. The immunosensor performed well in real blood samples, displaying a broad linear range and a low LOD for HER2 [123]. Prior research used high-density gold-coated silicon microneedle arrays as an EB transducer and an oncomarker extraction device to allow HER2 immunocapture and measurement. The device displayed a linear response in artificial interstitial fluid across a wide concentration range of 10 to 250 ng/mL [124].

Using functionalized ZIF-67 and ZIF-90, Xu et al. created a simple ratiometric EB aptasensor for the sensitive detection of HER2. The ZIF-67@Fc/antimonate nanoflakes capture probe had a high specific surface area, high conductivity, and the ability to bind aptamer single-stranded deoxyribonucleic acid (ssDNA). So when aptamer ssDNA came into contact with the oncomarker HER2, it was immediately desorbed from its surface. The signal probe, ZIF-90@MB, enabled one-step encapsulation of the MB signal while preventing interference from the surrounding environment. Once the capture probe recognized the target-HER2, the electrode conductivity decreased [125].

Laser-scribed graphene (LSG) has demonstrated tremendous promise as a sensing substrate. Lahcen et al. suggested a biosensing platform based on LSG electrodes that had been nanostructured with gold and MIP. A polyimide sheet was irradiated with a CO_2_ laser to make LSG electrodes. Gold with a nanostructure was electrodeposited onto the LSG to increase sensitivity and HER2 immobilization on the sensor surface. After 20 min of HER2 pre-adsorption on the electrode surface, 3, 4-ethylene dioxythiophene was electropolymerized to create MIP. They investigated and enhanced MIP deposition, removal, and adsorption properties. In spiked, undiluted human blood samples, the biomimetic sensor demonstrated significant HER2 recovery values and good selectivity for detecting HER2 in other interfering chemicals [126].

#### 4.1.7. EGFR

**Figure 3:**
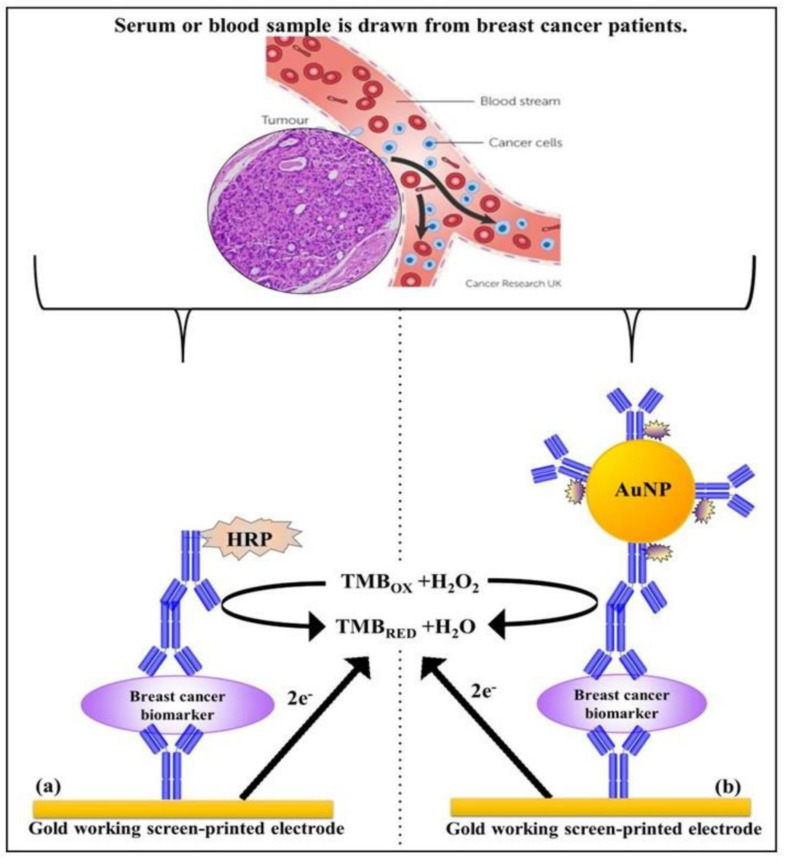
Fabricated chips are shown for the indirect and the indirect AuNPs enhanced sandwich biosensor for HER-1 and HER-2 oncomarkers; (**a**) standard assay and (**b**) AuNP assay [127].

In a study, two immunosensors were developed using gold sensor chips coupled with horse radish peroxidase (HRP) enzyme using an amperometric method by Wignarajah et al. [127]. These sensors are based on indirect sandwich ELISA assays using monoclonal antibodies (Ab) against HER-1 and HER-2 oncomarkers. The analytical results demonstrated satisfactory LOD and limit of quantification (LOQ) in the buffer and serum. For increasing sensitivity and specificity and decreasing cross-reactivity, AuNPs labeled with secondary IgG Ab-HRP were then developed (Figure 3). These findings show early detection and monitoring of disease progression in clinical outcomes.

#### 4.1.8. EpCAM

Jalil et al. synthesized molybdenum disulfide (MoS2) grafted reduced graphene oxide (MoS_2_@rGO) nanohybrid as an ultrasensitive electrochemical biosensor electrophoretically deposited on indium tin oxide coated glass substrate. Then, anti-EpCAM antibodies have been covalently immobilized on the MoS_2_ @rGO/ITO electrodes for EpCAM oncomarker detection in the 0.001–20 ng/mL concentration range. This platform offers an analytical method for monitoring the EpCAM oncomarker [128].

### 4.2. Circulating Tumor Cells (CTC)

As a “liquid biopsy”, the study of CTCs during the early stages of cancer helps patients be monitored at various periods in the disease cycle, including little residual disease, providing significant insights regarding the early assessment of treatment success.

The researchers employed non-spherical AuNPs electrodeposited in the presence of ethosuximide as a shape-directing and size-controlling agent. NPs with roughness ratings of 8.03 and sizes ranging from 50 to 150 nm covered the underlying surface. The apta-cytosensor transducer was then built using non-spherical NPs. The transducer surface was coated using an 83-mer DNA aptamer optimized for capturing cell surface proteins and binding with the cells and evaluated using the ferro/ferricyanide redox marker. After 200 min, the aptamer was immobilized on the transducer surface [129].

**Figure 4:**
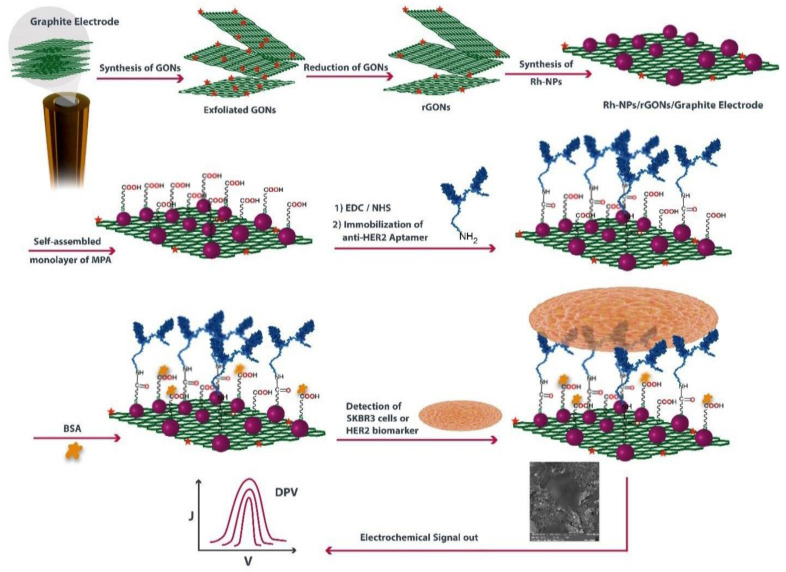
The fabrication process of aptasensor for detecting HER2 oncomarker: (i) the synthesis of rGO nanosheets, (ii) the synthesis of rhodium NPs, (iii) the immobilization of anti-HER2 aptamer strands, and (iv) the detection of HER2+ BC cell using the proposed aptasensor [131].

Han and colleagues explained developing an antifouling EB that can directly monitor CTCs in blood using a multifunctional peptide and an electrodeposited conducting polymer (PEDOT). The proposed peptide could trap MCF-7 BC cells and was antifouling in complicated biological conditions. Electrodeposited PEDOT enhanced electron transport at the sensing surface, boosting the sensitivity of the biosensor by enhancing the signal-to-noise ratio for detection. The suggested biosensor could work instantly in blood samples due to the multifunctional peptide and the conducting polymer PEDOT. For detecting MCF-7 cells, the antifouling EB had a large linear range spanning four orders and an LOD of 17 cells/mL. This paper proposed a viable approach for the rapid and painless direct identification of CTCs in human blood, which might be useful in cancer liquid biopsies [130]. We created a hybrid nanocomposite by combining rGO nanosheets with rhodium NPs on the surface of a graphite electrode (Figure 4). The graphite electrode-based aptasensor outperformed in SKBR3 cancer cells. Once folded into an intermolecular G-quadruplex, the G-rich DNA aptamers are precisely attached to the target molecule [131]. 

An xFe_2_O_3_-nPt@graphene (nPt-xFe_2_O_3_)-coated graphene nanostructure was effectively produced to use a superior atomic layer deposition approach. It has been demonstrated that graphene may acquire tailored deposits of xFe_2_O_3_ and nPt. A simple, quick, and sensitive cytosensor based on programmable NMs was successfully built to detect MCF-7 cells by applying an aptamer with high affinity and specificity. The built cytosensor responded linearly to MCF-7 concentrations [132]. Regarding peptides’ numerous appealing and desirable properties, attention to electrochemical biosensing has increased. In response to target binding events, peptides emit an audible electrical signal. The target-recognition and self-assembly competent amphipathic peptide FFFGGGGRGDS was engineered to co-assemble with the electroactive species ferrocene carboxylic acid. In the sensitive EB assessment of tumor cells, electroactive peptide nanoprobes were utilized (ePNPs). ePNPs preferentially attached to integrin proteins on the cell surface once tumor cells were captured using an electrode modified with the appropriate DNA aptamers, resulting in a considerable increase in EB signal [133].

Herceptin-conjugated graphene biosensors to identify HER2-positive BC cells were made easy and affordable by Rahimzadeh et al. The bifunctional graphene-Herceptin nanosheets were synthesized from graphite using a straightforward ultrasonic-mediated method. The produced protein-mediated graphene has undergone extensive characterization. The findings demonstrated that graphene layers peeled apart in the presence of Herceptin. The developed Herceptin-conjugated graphene was employed in the detection of BC. A wide linear range of 1–80 cells was available for this biosensor. The biosensor was effective in detecting cancer cells that are HER2-positive. The stability and function of the biosensor lasted for around 40 days. According to the results, this method seems to be a strong competitor for rapidly and accurately identifying cancer cells [134].

mXenes are a distinct class of conductive two-dimensional materials that have acquired prominence in biosensing due to their high surface area and distinctive surface chemistry. In recent work, mXene nanosheets with a thickness of around 2 nm and a lateral dimension of 1.5 m were attached to gold electrodes to detect tumor cells. On the mXene layers, electrostatic interactions trapped the HB5 aptamer with excellent selectivity for HER2-positive cells. To avoid electrode biofouling with the blood matrix, magnetic nanohybrids of CoFe_2_O_4_@Ag connected to the HB5 were used to separate HER2-positive CTCs. Sandwich-like structures formed by magnetically confined cells and functionalized mXene electrodes effectively protected the electron transport of a redox probe, allowing for current change-based quantitative cell detection. Researchers were blown away by the label-free mXene-based cytosensor device, which had a low LOD of 47 cells/mL and a broad linear range of 10^2^–10^6^ cells/mL. The proposed aptacytosensor had a high potential for tracking the progress of cancer cells in the blood by employing low-cost CoFe_2_O_4_@Ag magnetic nanohybrids and mXenes [135].

**Figure 5:**
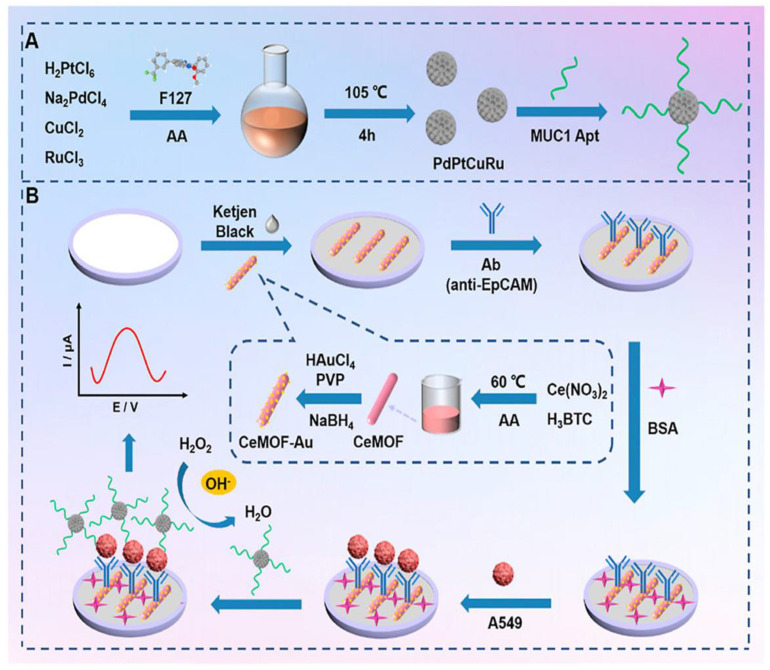
(**A**) The synthesis procedure of PdPtCuRu MNSs. (**B**) Construction of the sandwich-type electrochemical biosensor [136].

In a study by Zhou et al., a dual-recognition electrochemical biosensor for CTC detection was designed to better the signal response and specificity (Figure 5). PdPtCuRu mesoporous nanospheres were synthesized and connected to mucin 1 (MUC1) aptamer as a signal amplification probe and superconductive carbon black-AuNPs modified CeMOF-Au as a signal transducer. The designed biosensor showed good specificity and accuracy with LOD of less than 10 cells/mL in spiked serum samples [136].

### 4.3. Circulating Tumor-Specific DNA (ctDNA)

A promising noninvasive approach for identifying cancer in various malignancies, including BC, is ctDNA, sometimes called “liquid biopsy”. The applications of molecular analysis of ctDNA in BC have increased without considering the issue of tumor heterogeneity, including prognosis prediction, acquired resistance, response to therapy, and diagnosis. The ctDNA molecule as a biomarker in peripheral blood can be easily used for cancer diagnostics and therapeutics. The concentration of ctDNA is from 0 to 100 ng/mL, with an average of 30 ng/mL for healthy people, while ctDNA concentration ranges from 0 to 1000 ng/m, with an average of 180 ng/mL in the blood of cancer patients [137].

Polyetherimide-fragment crystallizable (PEI-Fc) and polyacrylic acid self-assembled on SiO_2_ NPs to form hollow polymeric nanospheres with high ferrocene loading efficiency. Fc-HPNs were used as an effective EB tag, and a dual-enzyme-assisted target amplification approach was applied to create an ultrasensitive EB for the detection of ctDNA. The high Fc tag loading on the hollow polymeric nanospheres and the catalytic activity of ascorbic acid in the Fc reduction process significantly enhanced the EB signals. With a low LOD of 1.6 fM and a fine linear response to ctDNA concentrations ranging from 10 fM to 10 nM, the proposed biosensor offers tremendous promise for cancer detection and therapy [138].

Zhao et al. offered a new nanocomposite MWCNT–PDA–Au–Pt by uniformly dispersing Au–Pt alloy NPs on MWCNT–PDA. The MWCNT–PDA–Au–Pt had a good reduction effect on H_2_O_2_ and could amplify the current response. It could be mixed with signal probes (SPs) and 6-mercapto-1-hexanol (MCH) to form SPs-label used to construct the EB. The capture probe (CPs) was fixed on the surface of the SPGEs by Au–S bond. Both SPs and CPs are DNA. CPs recognized and captured the target ctDNA. Then, SPs-label was added to form a sandwich structure by base pairing. The ctDNA samples had been linked to TNBC. When the target ctDNA was detected, the current characteristics changed significantly, and the corresponding current was lower than the current for undetected ctDNA. The linear detection range of the ctDNA nano biosensor was from 1 × 10^−15^ mol/L to 1 × 10^−8^ mol/L, and the LOD was as low as 5 × 10^−16^ mol/L [139].

In a study, high-active carbon (HAC) and AuPt alloy NPs were combined to form nanocomposites used as a signal amplification label to construct a sandwich-type ctDNA EB. The size distribution of HAC was uniform, and AuPt alloy NPs were effectively loaded onto HAC. SPs-label was generated by assembling the nanocomposites with DNA SPs using Au-S or Pt-S. The target DNA (tDNA) and SPs-label were incubated sequentially on the CPs-modified electrode after the capture probes (CPs) were immobilized on the electrode surface [140]. The researchers developed a new EB for ctDNA assays that combines wheel-like catalytic hairpin assembly (WCHA) and frame hybridization chain reaction (FHCR). Moreover, the metastable dumbbell probes (DSH) 1 and 2 were created for hybridization chain reaction [141].

### 4.4. Circulating miRNAs

MicroRNA molecules are noninvasive oncomarkers present in human body fluids such as blood, lymph, milk, tears, urine, and cerebrospinal liquid for early diagnosis of various cancers [142]. Pimalai et al. modified an electrode with rGO/poly(2 amino benzylamines)/AuNPs and employed porous, hollow silver gold NPs as metal ion tagging to generate extremely sensitive EB for miRNA detection. Furthermore, an anti-DNA-RNA hybrid antibody was developed employing distinct hybridized capture DNAs and miRNAs that can detect several miRNAs at the same time. The devised EB platform demonstrated outstanding performance with the range of 1 fM to 10 nM and a low LOD of 0.98 fM, 3.58 fM, and 0.25 fM for miRNA-155, miRNA-21, and miRNA-16 [143]. Bharti et al. demonstrated an EB for miRNA-21 hybridization detection based on a gold platinum bimetallic NPs (AuPtBNPs)/3-aminopropyltriethoxy silane nanocomposite covered with fluorine-doped tin oxide. The biosensing device achieved an LOD of 0.63 fM with a broad linear range, i.e., 1 fM-100 nM, for miRNA-21 detection [144].

Zhang et al. developed a novel microRNA EB based on 2D NMs composed of antimonide nanoflakes and carbon quantum dots to detect the BC-relevant oncomarker microRNA-21. MicroRNA-21 could be detected at concentrations ranging from 100 aM to 1 nM, with an LOD of 21 aM, which was three times lower than the existing microRNA biosensors [145]. They proposed an exo-miRNA measuring biosensor based on multifunctional DNA tetrahedron-assisted catalytic hairpin construction (MDTs-CHA) [146].

According to the study conducted by Lin et al., interactions between streptavidin (SA) and biotin in biotinylated detection probes (biotin-DNA-biotin) resulted in the in situ formation of tetrameric SA proteins on an electrode surface. The probe was divided into tiny pieces (biotin-DNA) after hybridizing with the target miRNA by the duplex-specific nuclease. The released target miRNA joined the next cycle of hybridization–enzymolysis, forming a large number of biotin-DNA fragments. Due to the competition for SA binding between the released biotin-DNA and the detection probe, in situ creation of (SA-biotin-DNA-biotin) assemblies was limited [147].

Farshchi et al. developed a novel paper-based EB peptide nucleic acid sensor for detecting miRNA-21 in human plasma samples by electroplating Ag@Au core–shell NPs on graphene quantum dots conductive nano-ink [148]. Zhu et al. created a new co-catalytic ferrocene/hemin/G-quadruplexes/Fe_3_O_4_ nanocomposite. The created EB had an LOD of 74.8 aM and could detect miRNA-155 at extremely low concentrations between 0.1 fM and 1 nM [149].

Pothipor et al. reported the addition of a nanocomposite of graphene, polypyrrole, and AuNPs to a screen-printed carbon electrode to improve electron transport properties and increase the degree of MB intercalation for signal amplification. The excellent EB reactivity of the GP/PPY-modified electrode and the high dispersibility of AuNPs contributed to good sensor performance [150].

Zayani et al. described a new method to sensitively determine miRNA levels via dual signal readout involving competitive hybridization between the miR-21 target and its biotinylated analog towards the same thiolated DNA probe attached to the surface of AuNPs. Hybridization of the DNA probes by the biotinylated miRs followed by conjugation with streptavidin-HRP catalyzed the oxidation of phenylenediamine into 2,3-diamino phenazine, detecting the target by fluorescent and EB methods. The two signals varied in the miRNA concentration-dependent manner. The bioplatforms had LODs of 15 and 19 fmol/L (0.15 and 0.19 attomol in 20 µL) [151].

A versatile electrochemical biosensing device was created for miRNA detection by applying DNAzyme cleavage cycle amplification and hybridization chain reaction (HCR) amplification. DNA-walker employed mn^2+^-focused DNAzyme cleavage to follow predesigned DNA tracks. Several G-quadruplex-incorporated long double-stranded DNA (dsDNA/G-quadruplex) complexes were generated for the EB technique. The G-quadruplex approach enables sensitive and precise miRNA quantification down to 5.68 fM [151]. 

Kim et al. offered an approach for a label- and wash-free EB miRNA detection with zeptomolar sensitivity based on target miRNA-induced Cu^2+^ reduction and subsequent changes in signals created from the residual Cu^2+^. Target miRNA was successfully detected utilizing this core approach with a detection limit of 33.2 zM. The synergistic coupling of the Cu^2+^ reduction and miRNA recycling processes enabled the ultrasensitivity. The utility of the proposed technique was proven by accurately identifying the target miRNA in total RNA samples acquired from diverse cancer cell types [152].

An EB microRNA biosensor was built on an antimonide quantum dot to detect BC-relevant biomarkers such as microRNA-155 and microRNA-21. As it possessed a more delocalized 5s/5p orbital, the antimonide had a stronger force interaction with single-strand RNA than graphene, according to the first principle of energetic computation. Double-stranded RNA had a low affinity for antimonide, making it simple to desorb the hybridized target from the antimonide interface using complementary microRNAs. MicroRNA-21 and microRNA-155 concentrations could be identified from 0 to 1 pM with low LOD from 64 to 89 aM. [153]. In a study by Tian et al., a ratiometric electrochemical biosensing device for miRNA detection was constructed by integrating bipedal DNAzyme walker breakage cycle amplification with planar intercalated MB molecule amplification. The sensitivity of the ratiometric biosensing technique and practicality was demonstrated by utilizing miRNA-21 as a triggered model target from MCF-7 cells and HeLa cells. This was accomplished using a cleaved Zn^2+^-dependent DNAzyme, which boosted the duplex section of the MB signal while decreasing the streptavidin-conjugated cupric sulfide@platinum nano zyme signal. The proposed sensing method for increased cleavage activity of the bipedal DNAzyme walker cyclic amplification produced an unexpectedly sensitive test for miRNA-21 [154].

A novel nano biosensor was fabricated based on dual signal-labeled hairpin-structured DNA (dhDNA) probes. The outside reference probe was a ferrocene-modified anti-miRNA-21 DNA probe, whereas the interior reference probe was a hairpin capture probe tagged with thiolated methylene blue (MB-HCP). An integrated structure comprising MB-HCP and Fc-AP-21 was built on a single sensing interface in a single experiment to detect miRNA-141 and miRNA-21. The dhDNA was first adsorbed onto the altered GCE to create the biosensor. The final structure of the biosensor was formed by pushing the dhDNA structure to open during hybridization with the anti-miRNA-141 complementary sequence (ACP-141). Further, miRNA-141 and miRNA-21 broke down the complexes because of the well-matched sequences between miRNA-141 and ACP-141 and miRNA-21 and Fc-AP-21 [155].

A label-free EB for miR-21 measurement was developed effectively. This biosensor boosted the MXene-MoS_2_ heterostructure and used the catalytic hairpin assembly (CHA) amplification approach. The innovative micro–nano heterostructure showed impressive confinement effects due to its huge specific area and strong electroconductivity. As a result, the original CHA amplification strategy was revived by the MXene-MoS_2_ heterostructure by activating additional target recycling. The MXene-MoS_2_ heterostructure surface was also coated with AuNPs and thionine, allowing the sensor to fixate capture probes and detect whether miR-21 was labeled. The design, therefore, revealed a dynamic range equal to or less sensitive than previously described methods for miR-21 detection [156].

### 4.5. Extracellular Vesicles (EVs)

EVs comprise apoptotic bodies, exosomes, and microvesicles. They range in size from 30 to 3000 nm and are released upon cellular activation, apoptosis, and senescence. A growing body of data suggests that EVs can facilitate specialized cell-to-cell communication [157]. 

Zhang and colleagues produced K_4_[Fe(CN)_6_]^3^ on Ti_3_C_2_ MXene as hybrid nanoprobes to build a sensitive EB to discover exosomes and their surface protein. To interact with the CD63 protein on exosomes generated by OVCAR cells, a CD63 aptamer-modified poly(amidoamine)-AuNP electrode interface was designed. Due to the twofold amplification effect, exosomes were detected electrochemically in a very sensitive and selective way (range: 5 × 10^2^ to 5 × 10^5^ particle μL^−1^, LOD:229 particle L^−1^) [158].

**Figure 6:**
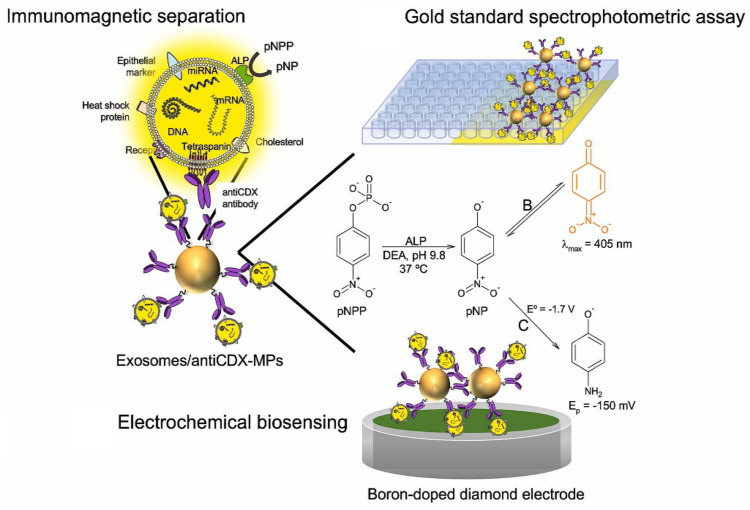
Different approaches for detecting ALP activity in osteoblast-derived exosomes by optical and electrochemical biosensors [159].

Moura et al. created an EB that combined immunomagnetic separation with biosensing based on the exosome’s intrinsic alkaline phosphatase (ALP) activity. The intrinsic activity of ALP in exosomes was investigated as a potential predictor of osseous metastatic invasion and carcinogenesis. For the first time, they explored two types of oncomarkers on exosomes by combining two distinct biorecognition reactions—immunological and enzymatic—in a unique biosensing device. Human fetal osteoblast exosomes were used as an in vitro model. With an LOD of 4.39 mU/L, similar to 10^5^ exosomes μL^−1^, the EB improved the analytical performance of the gold standard colorimetric test for detecting ALP activity in exosomes. By this approach, exosomes derived from blood samples of BC patients were quantified (Figure 6). Based on immunomagnetic separation employing particular epithelial oncomarkers EpCAM in combination with the intrinsic ALP activity, the EB accurately distinguished between healthy donors and BC patients [159]. 

Hashkavayi et al. proposed a screen-printed carbon electrode modified with a multi-walled CNT (MWCNT), IL, and Cs composite electrodeposited by AuNPs (GNPs/MWCNT-IL-CHT/SPCE) for the detection of exosome biomarkers. The exosome surface protein specific for CD63 was immobilized on the mentioned structure. These proposed analysis systems could simultaneously detect EpCAM- and HER2-positive exosomes with high specificity and a detection limit of 1 particle/mL [160]. In Table 1, a number of other methods are presented in the field of detecting exosomes from breast cancer cells, the results of which indicate that the electrochemical detection method can compete with them.

## 5. Perspectives

Tremendous advances have been made in the field of nano biosensors. However, researchers are facing many challenges in this field. The most important prevalent issues are developing reproducible calibration methods, applying preconcentration and separation methods, and integrating the nano biosensor with other elements of a sensor package in a reliable manner. Moreover, finding a suitable technology while maintaining the highest sensitivity and specificity is another problem. The ultimate goal of numerous studies is to design small biosensors with the lowest detection limit and detection time, consuming less energy and cost.

## 6. Conclusions

Despite progress in the last decade, traditional medical diagnoses for monitoring disease stages are still hindered by grand challenges such as lack of POC diagnostic devices, the need for expertise, low sensitivity, and selectivity resulting in false positive results. The development of POC diagnostic tools for BC oncomarkers is highly needed. This review emphasized the importance of specific BC oncomarkers and nanomaterials, including metal nanoparticles and graphene derivatives, for developing electrochemical nano biosensors for POCT owning to their simple operation, valid performance, and easy miniaturization and portability in cancer detection. The use of ultrasensitive affinity-based electrochemical biosensors is one of the most promising candidates with unique potential properties for cancer detection, which will be the basis for future research on this disease.

## Figures and Tables

**Table 1:** Different biosensors for exosomal BC detection.

Biosensing Method	Oncomarker	LOD	Linear Range	Sample Type	Refs.
SERS	MUC1, HER2, and CEA	N/A	N/A	Exosome-spiked serum	[161]
SERS	EpCAM and HER2	1.5 × 10^2^ particles/mL	2.7 × 10^2^ to 2.7 × 10^8^ particles/mL	MCF-7 cell-derived exosomes	[162]
ECL	Mir-155	273.20 aM	1.0 fM to 1.0 nM	Serum	[163]
FO-SPR	HER2 and EpCAM	HER2: 7 × 10^8^ particles/mL and EpCAM: 1.1 × 10^8^ particles/mL	N/A	Plasma	[164]
SPR	TNC	18.1 particles/mL^−1^	3 × 10^4^∼3 × 10^7^ particles mL^−1^	Circulating exosomes in serum	[165]
EIS	HER2	10 pg	0.1 ng to 1 µg	Serum	[166]
Fluorescence	PD-L1	880 particles μL^−1^	0.0001.0 and 0.2.0 particles μL^−1^	Plasma	[167]
Electrochemical	EpCAM	4 × 10^2^ exosomes μL^–1^	N/A	Serum	[168]
(MFS)-CRISPR assay	miR-21	1.2 × 10^3^ particles/mL	10^4^–10^8^ particles/mL	Plasma	[169]
Fluorescence	CD63	2.5 × 10^3^ particles/μL	N/A	MCF-7 cell exosomes in FBS	[170]
Fluorescence	MUC1	7.56 particles/μL	0−400 particles/μL	Plasma	[171]

surface-enhanced Raman scattering (SERS); electrochemiluminescence (ECL); fiber-optic surface plasmon resonance (FO-SPR); electrochemical impedance spectroscopy (EIS); membrane fusion strategy (MFS); Mucin (MUC1); Tenascin-C (TNC); programmed death ligand-1 (PD-L1); fetal bovine serum (FBS).

## Data Availability

Not applicable.
